# Fatal anaphylaxis registries data support changes in the who anaphylaxis mortality coding rules

**DOI:** 10.1186/s13023-016-0554-4

**Published:** 2017-01-13

**Authors:** Luciana Kase Tanno, F. Estelle R. Simons, Isabella Annesi-Maesano, Moises A. Calderon, Ségolène Aymé, Pascal Demoly, on behalf of the Joint Allergy Academies

**Affiliations:** 1 Hospital Sírio Libanês, São Paulo, Brazil; 2 University Hospital of Montpellier, Montpellier, France; 3 Sorbonne Universités, UPMC Paris 06, UMR-S 1136, IPLESP, Equipe EPAR, 75013 Paris, France; 4 Section of Allergy & Clinical Immunology, Department of Pediatrics & Child Health, University of Manitoba, Winnipeg, Canada; 5 Section of Allergy and Clinical Immunology, National Heart and Lung Institute, Royal Brompton Hospital, Imperial College London, London, UK; 6 INSERM, US14, Paris, France

**Keywords:** Anaphylaxis, Classification, International Classification of Diseases, Mortality, World Health Organization

## Abstract

Anaphylaxis is defined as a severe life-threatening generalized or systemic hypersensitivity reaction. The difficulty of coding anaphylaxis fatalities under the World Health Organization (WHO) International Classification of Diseases (ICD) system is recognized as an important reason for under-notification of anaphylaxis deaths. On current death certificates, a limited number of ICD codes are valid as underlying causes of death, and death certificates do not include the word anaphylaxis *per se*. In this review, we provide evidences supporting the need for changes in WHO mortality coding rules and call for *addition of anaphylaxis as an underlying cause of death on international death certificates*. This publication will be included in support of a formal request to the WHO as a formal request for this move taking the 11^th^ ICD revision.

## Background

### Anaphylaxis definition and epidemiology

Definitions of anaphylaxis for clinical use by healthcare professionals all state the concepts of a serious, generalized, allergic or hypersensitivity reaction that can be life-threatening and even fatal [1]. In all countries, epidemiological and health services research can serve as a baseline for quality improvement, prioritization of anaphylaxis programs, and eventual reduction in morbidity and mortality.

Publications on anaphylaxis epidemiological data have increased in the past few years due to the need to understand the status and evolution of this disease more precisely worldwide, improve in order to plan national or global actions to support better management and prevention globally and nationally, and support education and awareness. Data can differ widely depending on a number of variables. For instance, European data have indicated incidence rates for all-cause anaphylaxis ranging from 1.5 to 7.9 per 100 000 person/year, with an estimation that 0.3% (95% CI 0.1–0.5) of the population will experience anaphylaxis at some point during their lifetime [2]. On the other hand, it is estimated that 1 in every 3000 inpatients in US hospitals suffer from an anaphylactic reaction with a risk of death around 1%, accounting for 500 to 1000 deaths annually in this country [3].

In public health terms, anaphylaxis is considered to be an uncommon cause of death [4–9]. The case fatality rate is difficult to ascertain with accuracy. Accurate anaphylaxis mortality data are hampered by the limited recognition of this condition among health professionals, the absence of historical details from eyewitnesses, incomplete death scene investigations, paucity of specific pathologic findings at postmortem examination, and the under-notification of anaphylaxis [9, 10].

## Vital statistics: historical background and current standard methods

The first International List of Causes of Death was drafted by Jacques Bertillon and colleagues in 1885. It was prepared based on the principle of distinguishing between systemic diseases and those localized to a particular organ or anatomical site, and officially adopted for use in mortality registries in 1893 [11]. This classification, which was accepted by many countries and has been periodically revised, constituted the basis of the International Classification of Diseases (ICD). Anaphylaxis was not included in the original list because it was not formally described until 1902 [12]. Although a well-known cause of death, particularly in the fields of allergy and emergency medicine, anaphylaxis has never been appropriately classified in the different versions of the ICD, and has never been considered an underlying cause of death on death certificates.

**Fig. 1:**
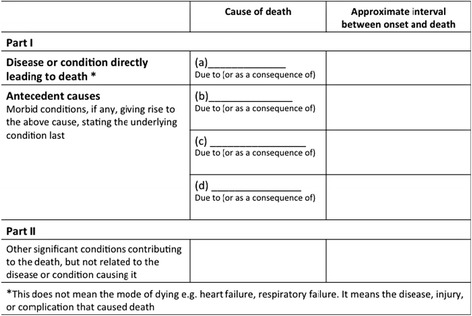
The World Health Organization’s International form of medical certificate of cause of death

Mortality statistics are widely used for medical research, monitoring of public health, evaluating health interventions and planning and follow-up of health care. Analysis of mortality data typically involves comparisons of data sets. However, unless the data have been compiled using the same methods and according to the same standards, comparisons potentially yield misleading results. For these reasons, the World Health Organization (WHO) issued international instructions on data collection, coding and classification, and statistical presentation of causes of death. In most countries, mortality statistics are routinely compiled according to regulations and recommendations adopted by the World Health Assembly (WHA). The international mortality coding instructions presuppose that data have been collected with a death certificate conforming to the *International form of medical certificate of cause of death* (Fig. 1) [13]. It is the responsibility of the medical practitioner or other qualified certifier signing the death certificate to indicate which morbid conditions led directly to death and to state any antecedent conditions giving rise to this cause.

The international death certificate form is split in 2 parts (Fig. 1). Part 1 is for diseases or conditions related to the sequence of events leading directly to death, and Part 2 is for unrelated but contributory conditions. The terminal cause of death is the condition entered first on the first line of Part 1 of the death certificate. The underlying cause of death is the condition selected for such single-cause tabulation. In most cases, the underlying cause of death is the same as the starting point of the sequence described in Part 1. Special coding instructions on specific sequences and ICD categories may have the effect that a condition other than the starting point is selected as the underlying cause of death for use in the vital statistics [13].

If an apparent error is found in the mortality data notification, it should be reported to the WHO, which will either explain the rationale or take steps to correct the error at the international level. Individual countries should not correct what is assumed to be an error, since changes at the national level will lead to data that are not comparable to data from other countries, and thus less useful for analysis [13].

## Anaphylaxis mortality data: unmet needs

### Allergic and hypersensitivity conditions in the ICD-11

Anaphylaxis mortality epidemiological data are sparse. Besides the different methods used and the different populations studied (Table 1), the lack of standardized definitions for this condition in the WHO ICD [1, 10, 14] is a recognized challenge for the development of accurate and comparable population-based vital statistics in the field.

**Table 1:** Anaphylaxis mortality publications, bold font highlights studies that utilized ICD registries as the basis for analysis

Reference	Study location	Study design	Number of subjects	Causes of anaphylaxis deaths (%)	Study period	Study fatality database	Current National Fatality database
Barnard et al. (1973) [29]	New York, United States of America	Retrospective case review	400	Hymenoptera sting/venom (100)	10 years (1966–1976)	Insect Sting Committee of the American Academy of Allergy, Asthma, and Immunology (AAAAI)	United States Vital Statistic Data
Bock et al. (2001) [30]	Denver and Yew York, United States of America	Retrospective case review with interview family members about the details of the fatality	32	Food-induced anaphylaxis (100)	5 years (1994–1999)	Food Allergy and Anaphylaxis Network and the AAAAI	United States Vital Statistic Data
Bock et al. (2007) [5]	Denver and Yew York, United States of America	Retrospective case review with interview family members about the details of the fatality	31	Food-induced anaphylaxis (100)	5 years (2001–2006)	Food Allergy and Anaphylaxis Network and the AAAAI	United States Vital Statistic Data
Delage et al. (1972) [31]	Washington D.C., United States of America	Retrospective case review with clinic-pathologic data analysis	43	Drug-induced anaphylaxis (100)	15 years (1957–1972)	Files of the National Armed Forces Institute of Pathology	United States Vital Statistic Data
Greenberger et al. (2007) [6]	Illinois, United States of America	Retrospective case review with clinic-pathologic data analysis	25	Pharmacological agents (52), hymenoptera sting (24), food (16)	12 years (1989–2001)	Office of the Medical Examiner of Cook County, Chicago, IL general mortality database	United States Vital Statistic Data
James et al. (1964) [32]	Boston, United Sates of America	Retrospective case review with clinic-pathologic data analysis	6	Pharmacological agents (84), hymenoptera sting/venom (16)	No data	Local database	United States Vital Statistic Data
**Jerschow et al. (2014)** [33]	**New York, United States of America**	**Population-based epidemiologic study using ICD-10 CM diagnostic codes on death certificates**	**2458**	**Medications (58.8), unspecified (19.3), venom (15.2), food (6.7)**	**11 years** **(1999–2010)**	**US National Mortality Database**	**United States Vital Statistic Data**
**Lenler-Petersen P et al. (1994)** [34]	**Copenhagen, Denmark**	**Retrospective case review based on ICD code (“collapsus anaphilaticus”) on death certificates**	**30**	**Drug-induced anaphylaxis (100)**	**22 years** **(1990–1968)**	**Danish Central Death Register**	**Danish Central Death Register**
**Liew et al. (2009)** [4]	**Melbourne, Australia**	**Retrospective case review based on ICD-10 codes on death certificates**	**112**	**Food (6%), drugs (20),** **probable drugs (38), insect stings (18),** **undetermined (13), other (5)**	**8 years** **(1997–2005)**	**National Hospital Morbidity** **Database and National Mortality Database**	**National Mortality Database maintained by the Australian Institute of Health and Welfare**
Low et al. (2006) [35]	Auckland, New Zealand	Retrospective case review with clinic-pathologic data analysis	18	Drugs (56), Hymenoptera sting (22), food (11), undetermined (11)	20 years (1985–2005)	Forensic Pathology Department database at Auckland City Hospital	Forensic Pathology Department database at Auckland City Hospital
**Ma et al. (2014)** [36]	**Virginia, United States of America**	**Population-based epidemiologic study using 3 national databases and selected ICD codes**	**186–225 deaths/year**	**Unspecified (66–85), drugs (11–27), food (4–7)**	**10 years** **(1999–2009)**	**Nationwide Inpatient Sample** **(NIS; 1999–2009), the Nationwide Emergency Department Sample (NEDS; 2006–2009), and Multiple Cause of Death Data (MCDD; 1999–2009)**	**US National Mortality Database**
**Mosbech H. (1983)** [37]	**Denmark**	**Population-based epidemiologic study based on ICD-8 code (E 905) on death certificates**	**26**	**Hymenoptera sting/venom (100)**	**20 years** **(1960–1980)**	**Danish Central Death Register**	**Danish Central Death Register**
Pumphrey et al. (2000) [38]	Manchester and Oxford, United Kingdom	Retrospective case review with clinic-pathologic data analysis	164	Drugs (37.5), hymenoptera (34), food (28.5)	6 years (1992–1998)	Office of National Statistics (ONS) database	UK Office of National Statistics (ONS) database
Sampson et al. (1992) [39]	Connecticut, United States of America	Retrospective series of cases study including food-induced anaphylaxis deaths in children and adolescents	6	Food-induced anaphylaxis (100)	14 months	Local database	US National Mortality Database
Sasvary et al. (1994) [40]	Switzerland	Retrospective series of cases study with clinic-pathologic data analysis	29	Hymenoptera sting/venom (100)	9 years (1978–1987)	Local database	Swiss National Mortality Database
Shen et al. (2009) [7]	Maryland (United States) and Shanghai (China)	Retrospective case review with clinic-pathologic data analysis	28	Drugs (57), food (21.5), unknown (10.7), hymenoptera (7.2), other (3.6)	3 years (2004–2006)	Office of the Chief Medical Examiner for the State of Maryland (OCME-MD) and the Department of Forensic Medicine at Shanghai Medical College (FM-SHMC)	US National Mortality Database
**Simon et al. (2008)** [41]	**Florida, United States of America**	**Population-based epidemiologic study based on ICD-9 and ICD-10 codes on death certificates**	**89**	**Drugs and radio contrast media (34), hymenoptera (12), food (6)**	**10 years** **(1996 to 2005)**	**Florida Department of Health, Office of Vital Statistics**	**US National Mortality Database**
**Tanno et al. (2012)** [10]	**São Paulo, Brazil**	**Population-based epidemiologic study based on ICD-10 codes on death certificates**	**498**	**Drugs (42), insect bite (35), unspecified (21), food (2)**	**3 years** **(2008–2010)**	**Brazilian Mortality Information System** **(SIM)**	**Brazilian Mortality Information System** **(SIM)**
**Turner et al. (2014)** [42]	**United Kingdom**	**Hospital admissions and fatalities caused by anaphylaxis data from national databases cross-checked against a prospective fatal anaphylaxis registry based on ICD-9 and ICD-10.**	**480**	**Drugs (54.8), food (25.8), insect sting (19.4)**	**20 years** **(1992–2012)**	**Office of** **National Statistics (ONS) database**	**UK Office of** **National Statistics (ONS) database**
Yilmaz et al. (2009) [8]	Istanbul, Turkey	Retrospective series of cases study with clinic-pathologic data analysis	36	Drug-induced anaphylaxis (100)	5 years (2001–2006)	Council of Forensic Medicine database in Istanbul, Turkey	The Council of Forensic Medicine
Yunginger et al. (1988) [43]	Rochester, United States of America	Retrospective series of cases study with laboratory investigation	7	Food-induced anaphylaxis (100)	16 months (1987–1988)	Local database	US National Mortality Database
Yunginger et al. (1991) [44]	Maryland, Florida, Virginia; United States of America	Prospective post-mortem case–control study with application of laboratory investigation protocol	19	Hymenoptera stings (47.3), foods (42.2), or diagnostic/therapeutic agents (10.5)	No data	Local database	US National Mortality Database

Causes of deaths are classified and grouped according to the ICD edition in use at the time, currently ICD-10 (and adaptations), and the information on vital statistics is collected using the international form recommended by the WHO. However, on the current death certificates, a limited number of ICD-10 codes are considered to be valid for representing underlying causes of death. As an example, research showed an under-notification of anaphylaxis deaths due to difficult coding under the ICD-10 using the Brazilian national mortality database, given that there are no anaphylaxis-specific ICD-10 codes which are considered valid for coding underlying causes-of-death [10].

Taking the window of opportunity presented by the ongoing ICD-11 revision, the under-notification of death data [10] triggered a cascade of strategic international actions supported by the Joint Allergy Academies and the ICD WHO governance [15–25] to update the classifications of allergic conditions for the new ICD edition. These efforts have resulted in the construction of the new “Allergic and hypersensitivity conditions” section under the “Disorders of the Immune system” chapter [21, 26].

Here, in order to deliberate the new frame and follow the ICD-11 revision agenda, we reviewed the forms on which anaphylaxis has been classified in the ICD and the published anaphylaxis fatalities data, particularly with regards to the methods used for death notification. We also propose modifications in the WHO mortality coding rules under the 11^th^ revision of the ICD context.

### Status of anaphylaxis in the ICD-10 and the ICD-11 Beta draft

**Fig. 2:**
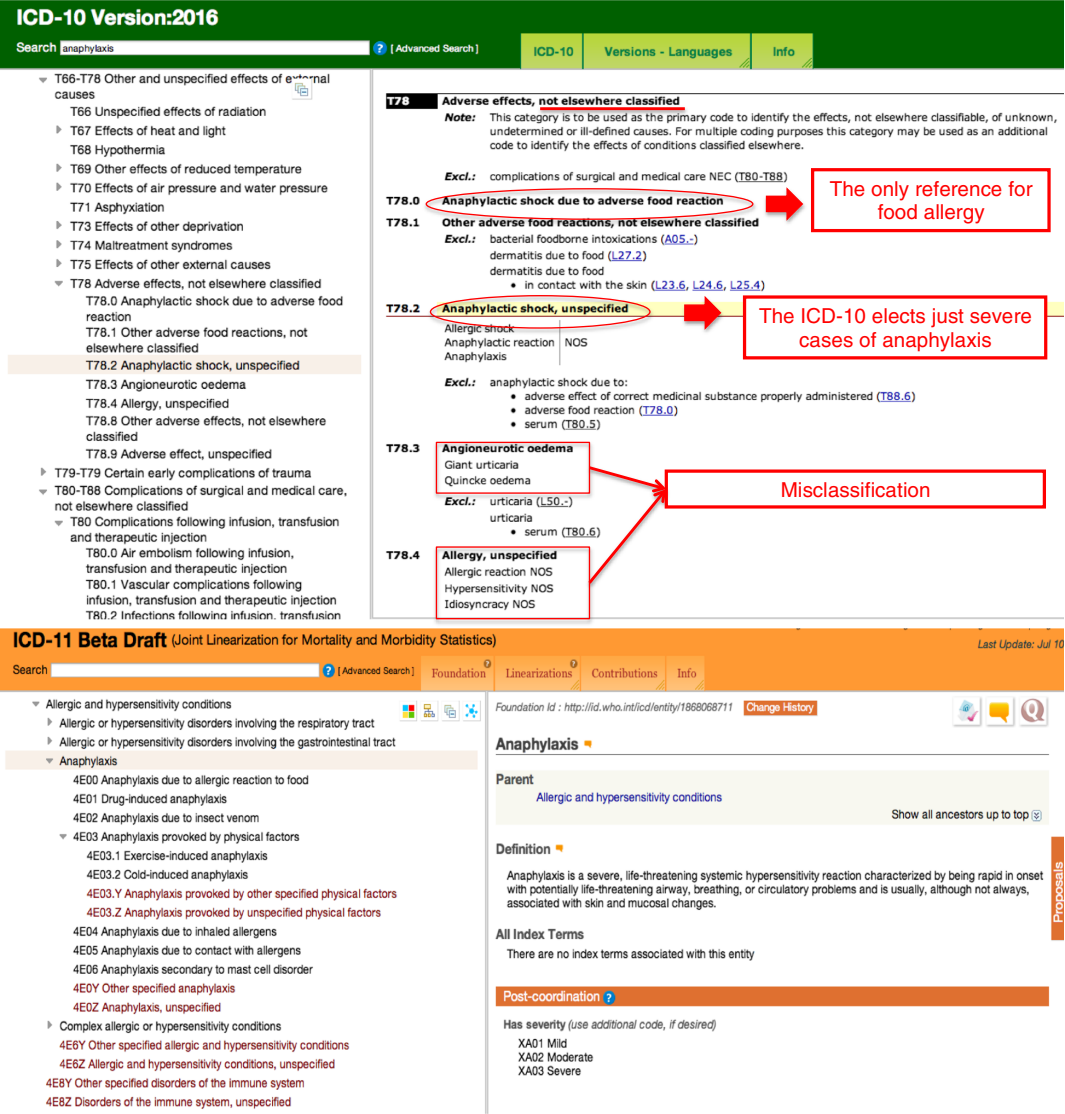
Anaphylaxis in International Classification of Diseases (ICD)-10 (2016 version) and ICD-11 Beta draft (July 2016 version). In *bold*, the headings of the ICD-10 T78 section and, in *red*, comments regarding misclassification of allergic and hypersensitivity conditions

The search for the term “anaphylaxis” in the online versions of the ICD-10 (2016 version) [27] and of the ICD-11 Beta draft Linearization (July 2016 version) [26] allows us to demonstrate the main differences resulted from all the efforts over the last 4 years (Fig. 2). The ICD-10 inherited the hierarchical scheme of the previous ICD versions, essentially based in main organs or main cause (infectious diseases or external causes). Therefore, some systemic conditions such as anaphylaxis were adjusted in the chapter related to external causes. As a result of our search into the ICD-10 (2016 version) platform, we have addressed the “XIX Injury, poisoning and certain other consequences of external causes” chapter, specifically the “T78 Adverse effects, not elsewhere classified” section. In Fig. 2 (highlighted in red) we also underline the lack of awareness of allergic and hypersensitivity concepts verified in the T78 section. Under this section it is possible to observe that only severe cases of anaphylaxis have been prioritized (T78.2 Anaphylactic shock), which was classified at the same level of “Anaphylactic shock due to adverse food reaction”, “Angioneurotic oedema” and “Allergy, unspecified”. In fact, obstruction of the upper and/or lower respiratory tract leading to respiratory distress and potential fatality is more commonly observed in anaphylaxis than hypotension and shock *per se*. It is also possible to note the misclassification implied in the ICD-10 exemplified by scattering “T78.2 Anaphylactic shock” at the same level of the “T78.3 Angioneurotic oedema”, “T78.4 Allergy, unspecified” and “T78.9 Adverse effect, unspecified” under the same heading (Fig. 2, in bold).

In the new “Allergic and hypersensitivity conditions” section of ICD-11, it was possible to build a sub-section specifically addressed to anaphylaxis. For the first time, anaphylaxis is elected as individualized conditions into the ICD-11 frame, receiving a sub-section addressed to this condition. Currently, this subsection contains 7 main anaphylaxis headings to be post-coordinated with severity and causality classification/specifications, still under tuning. The building block of this framework was the result of combined efforts and constant discussions with the groups of experts and the ICD WHO governance.

Based on the ICD-10 codes, some external stimuli are considered as underlying causes-of-death, but the word anaphylaxis as such has never been listed as an underlying cause-of-death. In fact, having allergic and hypersensitivity conditions classified in a more detailed scheme in the ICD-11 and not as in ICD-10 into a specific chapter in the “External causes of morbidity and mortality” or in the “Injury, poisoning and certain other consequences of external causes” chapters allows for capture of more realistic anaphylaxis mortality data from now on.

### What do the published fatal anaphylaxis data tell us?

Constructing a classification of anaphylaxis for ICD-11 was a challenge; however, it was important to align this with the published post-mortem anaphylaxis epidemiological data. From 30^th^ June 2015 to 4^th^ December 2015, thirty manuscripts were selected using PubMed Mesh terms “anaphylaxis deaths”, “anaphylaxis mortality”, “anaphylaxis fatalities”, covering documents published in the last five decades. We did not include case reports as such (with the exception of a few landmark case series), studies in animal models or reviews. All publications were independently evaluated by two co-authors and disagreements related to the inclusion into the analysis were resolved through open discussion. We analyzed methodological aspects, main outcomes and databases used in the remaining 22 publications (Table 1), 45% of which focused on specific triggers or etiology. Most of these documents (64%) were published over the last 15 years. The methods used and the population evaluated varied among the publications; however, 54.5% focused on US populations in different centers. Overall, 54% were based on national databases and 36.4% of these documents used the ICD for mortality registries as the basis of the analysis (as highlighted in bold in Table 1), with 62.5% being population-based studies. Based on ICD registries, regardless of the ICD version used, 87.5% of all the studies had to utilize secondary data in death certificates in order to capture the anaphylaxis data. Studies of anaphylaxis mortality using secondary data require the use of information derived from the underlying as well as the contributing cause-of-death. In other words, none of these deaths would have been found had the authors exclusively considered information from the underlying cause-of-death field.

## Conclusion

### Data support changes in the world health organization anaphylaxis mortality coding rules

In summary, in this manuscript, we provide evidence that supports the need for changes in the WHO mortality coding rules by adding *anaphylaxis as an underlying cause of death in international death certificates*. This article is a contribution to the establishment of ICD-11 to ensure a proper coding of anaphylaxis, in order to generate an accurate knowledge of the consequences of this severe condition. This document will comprise part of a formal request to the WHO to change mortality coding rules so that anaphylaxis can be listed as an underlying cause of death in international death certificates.

Once implemented by the WHO, there will be two immediate consequences of the use of the new classification based on the logic of the ICD-11: (i) although currently anaphylaxis fatalities are perceived as rare, the reported number of anaphylaxis deaths may increase [28] and (ii) most cases will be included in official mortality statistics, providing a global standard for comparability and, therefore, for decision-making and prevention.

## Abbreviations

ICD: International Classification of Diseases
WHA: World Health Assembly
WHO: World Health Organization
